# Thapsigargin triggers a non-apoptotic, caspase-independent programmed cell death in basophilic leukaemia cells

**DOI:** 10.1038/s41420-025-02602-w

**Published:** 2025-07-08

**Authors:** Philip Steiner, Korollus Melek, Ancuela Andosch, Lena Wiesbauer, Anna Madlmayr, Michelle Duggan, Hubert H. Kerschbaum, Susanna Zierler

**Affiliations:** 1 Institute of Pharmacology, Faculty of Medicine, Johannes Kepler University Linz, Linz, Austria; 2 Institute of Biochemistry and Molecular Medicine, University of Bern, Bern, Switzerland; 3 Department of Biosciences and Medical Biology, Paris Lodron University Salzburg, Salzburg, Austria; 4 Core Facility Imaging, Center for Medical Research, Faculty of Medicine, Johannes Kepler University Linz, Linz, Austria; 5 Walther Straub Institute of Pharmacology and Toxicology, Ludwig-Maximilians-Universität München, Munich, Germany

**Keywords:** Apoptosis, Nucleus, Endoplasmic reticulum, Super-resolution microscopy

## Abstract

Thapsigargin (TG), a potent inhibitor of the sarco/endoplasmic reticulum Ca²⁺-ATPase (SERCA), is widely used to study intracellular Ca²⁺ homeostasis and has shown—along prodrug derivatives—promise as an anticancer agent. While TG is traditionally considered an inducer of apoptosis, the precise mode of cell death it triggers remains incompletely defined. Here, we investigated the effects of TG on rat basophilic leukaemia (RBL-1) cells using advanced 2D and 3D transmission electron microscopy, confocal laser scanning microscopy, and functional cell death assays. TG treatment led to marked ultrastructural alterations, including pronounced ballooning of the perinuclear space, extensive vacuolization, mitochondrial enlargement and degradation, and structural anomalies of the endoplasmic reticulum. Notably, classical apoptotic features such as nuclear fragmentation, chromatin condensation and apoptotic body formation were absent. Functional assays revealed minimal caspase-3/7 activation and low Annexin V staining, indicating a caspase-independent, non-apoptotic form of programmed cell death (PCD). Morphological and quantitative analyses demonstrated that TG-induced cell death in RBL-1 cells closely resembles autosis, a non-apoptotic, autophagy-dependent PCD characterized by perinuclear space ballooning and increased autophagolysosome formation. These autosis-like features were also observed in TG-treated murine macrophages and human mast cells, suggesting a conserved mechanism across cell types. Digoxin, a Na⁺/K⁺-ATPase inhibitor, partially reversed TG-induced ultrastructural damage, supporting the involvement of Na⁺/K⁺-ATPase in this process. Ca²⁺ imaging confirmed that TG-induced cytosolic Ca²⁺ elevation is primarily driven by ER Ca²⁺ release, with extracellular Ca²⁺ amplifying the response. Our findings establish that TG induces a non-apoptotic, caspase-independent PCD matching autosis, challenging the prevailing view of TG as a classical apoptosis inducer. This insight has important implications for research on intracellular Ca^2+^ homeostasis as well as for the therapeutic exploitation of TG and its derivatives in targeting apoptosis-resistant cancer cells.

## Introduction

Thapsigargin (TG) increases free cytoplasmic Ca^2+^ upon selective inhibition of the sarco/endoplasmic reticulum Ca^2+^-ATPase (SERCA) pump [1] and subsequently activates cell death-promoting signalling cascades [1–5]. Inhibition of the SERCA pump by TG induces ER stress as seen by the unfolded protein response (UPR) [1–3]. Accumulation of unfolded proteins in the ER leads to the activation of the three ER stress sensors, PERK, IRE1 and ATF6 [1, 6]. As TG activates these ER stress sensors and UPR triggers the activation of downstream pro-apoptotic pathways, including the activation of caspase-12, caspase-9 and caspase-3, as well as the release of cytochrome c and the dissipation of mitochondrial membrane potential [4, 5], TG is considered as an inducer of an apoptotic pathway [1–3].

The identification of TG-induced cell death mechanisms is not only crucial for basic research but also holds important clinical relevance. Understanding the specific cell death modality activated in various pathological conditions is crucial for developing targeted therapeutic strategies, as different cell death pathways may have distinct impacts on inflammation, immune response or tissue homeostasis [7]. TG and respective precursor compounds have shown promising anticancer activity by selectively inducing apoptosis in tumour cells [1, 8, 9]. Recent studies have also highlighted the potential of TG-based prodrugs, such as mipsagargin, which can be selectively activated in the tumour microenvironment, further enhancing the therapeutic window [10, 11]. In the context of metabolic disorders, such as diabetes, TG has been suggested to induce apoptosis in pancreatic β-cells, underscoring the importance of ER homeostasis in the regulation of insulin secretion and glucose metabolism [2, 12, 13]. Moreover, TG has been found to synergize with other apoptosis-inducing agents, such as chloroquine, which inhibits autophagy and enhances apoptosis by increasing the Bax/Bcl-2 ratio. This underscores the importance of understanding TG’s mechanism of action, as it may enhance the efficacy of combination therapies in clinical settings, particularly given that the specific mode of cell death activated in cancer cells can significantly impact the overall therapeutic outcome.

Recent research has identified several novel cell death modalities with distinct stimuli, mechanisms and morphologies [14–16]. These new modalities often overlap with traditional categories but are distinct enough to necessitate a refined classification. Programmed cell death (PCD) is regulated by intracellular signals, while non-PCD results from unexpected cell injury. PCD can be further divided into apoptotic (caspase-dependent) and non-apoptotic (caspase-independent) types [16]. Apoptosis is characterized by cell shrinkage, membrane blebbing, organelle loss, DNA condensation and nucleus fragmentation. It is triggered through three main pathways: extrinsic (death receptors), intrinsic (mitochondrial) and perforin/granzyme pathways [17]. Anoikis, a subtype of apoptosis, occurs due to inadequate or inappropriate cell-matrix interactions, involving similar pathways but is influenced by cytoskeletal architecture and integrin function [18].

Recent studies have highlighted the critical role of Ca^2+^ homeostasis in regulating autophagy and non-apoptotic autosis, revealing a complex interplay between Ca^2+^ signalling and these cellular processes [19, 20]. Ca^2+^ serves as a second messenger that can both promote and inhibit autophagy, depending on the context, with specific Ca^2+^ channels and signalling pathways implicated in these effects [21, 22]. Dysregulation of Ca^2+^ levels can lead to impaired autophagic flux, contributing to various pathological conditions, including neurodegenerative diseases and cardiac dysfunction, underscoring the importance of maintaining Ca^2+^ homeostasis for cellular health and survival [23, 24]. Autosis is an autophagy-dependent, non-apoptotic form of cell death characterized by the ballooning of the perinuclear space due to excessive accumulation of autophagolysosomes [25]. It is induced by autophagy-inducing peptides, starvation, and hypoxia [26]. Additional unique features of autosis include very mild chromatin condensation, mitochondrial dilatation, increased vacuolation and its dependence on Na^+^/K^+^-ATPase, distinguishing it from other cell death pathways [25, 27]. A long-standing controversy in cell biology is whether autophagy directly causes mammalian cell death. Using the autophagy-inducing peptide Tat-Beclin 1, Liu, Shoji-Kawata [28] demonstrated that high levels of autophagy result in autosis. Their findings identified the Na^+^-K^+^-ATPase as a critical regulator of autosis and revealed that cardiac glycosides can inhibit this form of cell death, offering potential therapeutic insights for conditions involving cellular stress.

As elevated cytoplasmatic free Ca^2+^ not only triggers apoptosis but also autosis, we hypothesize that TG induces a non-apoptotic, caspase-independent form of PCD in rat basophilic leukaemia cells (RBL-1), characterized by distinct morphological changes that differ from the classical apoptotic pathway. To evaluate our hypothesis, we used advanced microscopy techniques, quantified caspase dependency of TG-induced PCD in RBL-1 cells, evaluated intracellular Ca^2+^ regulation and compared the morphological and physiological markers of TG-induced PCD in RBL-1 cells with those of known apoptotic and non-apoptotic PCD modalities. In addition, key experiments were extended to other cells of the innate immune system in order to get a first impression of the generality of our hypothesis.

## Results

### Thapsigargin treatment decreases nuclear HOECHST fluorescence but does not affect mitochondria, ER and endocytosis during RBL-1 live cell imaging

**Fig. 1:**
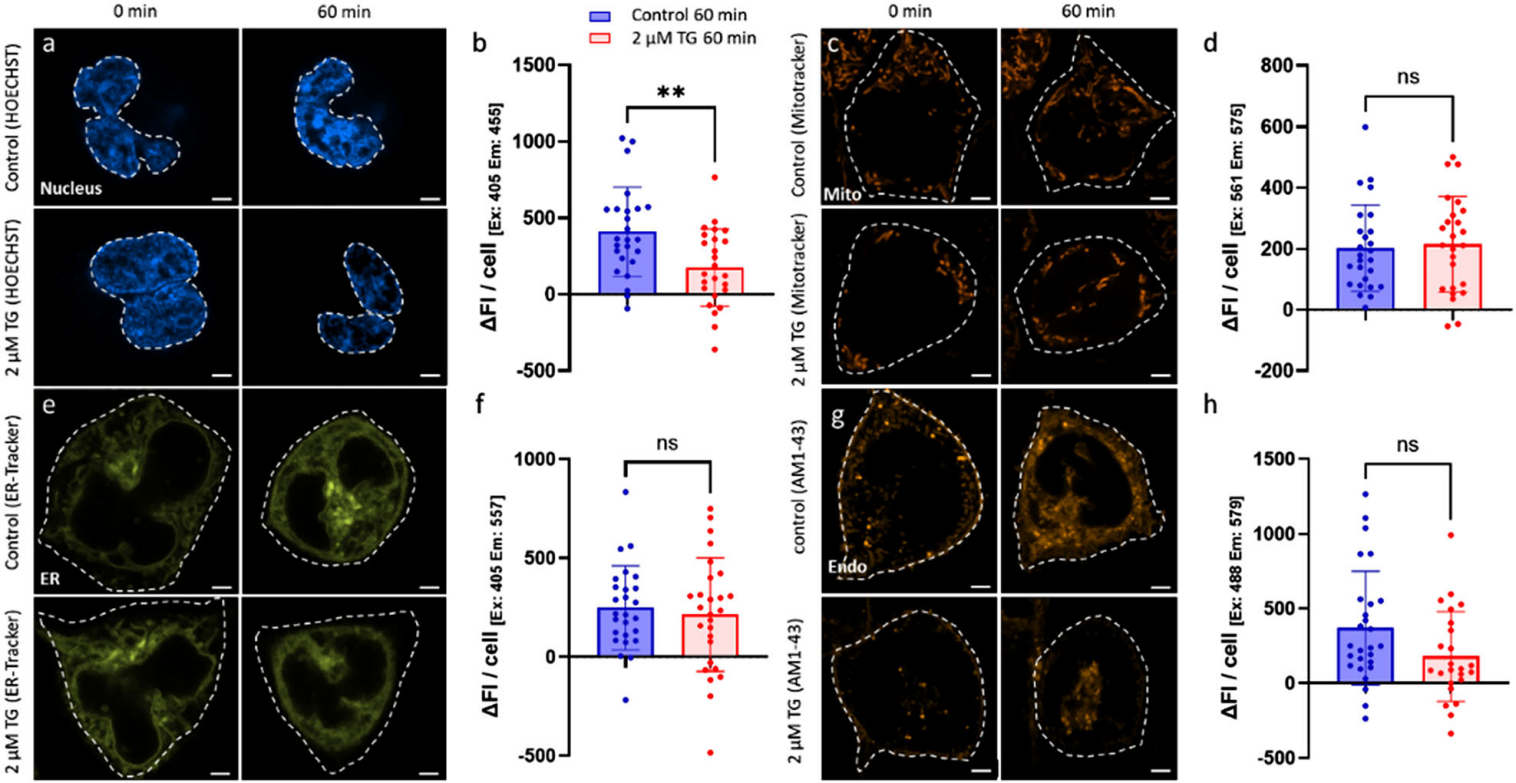
Confocal laser-scanning microscopic (CLSM) live cell imaging of different intracellular compartments in RBL-1 cells before (control) and after TG treatment over a treatment time of 60 min. **a** Representative images of HOECHST-labelled nuclei for control (upper row) and TG-treated cells (lower row) at the beginning (0 min) (left) and after 60 min (right). **b** Statistical comparison of the HOECHST fluorescence intensity change (ΔFI) over 60 min (corresponding to ROI) between control and TG-treated cells. *n* = 25. **c** Representative MitoTracker staining of mitochondria in control (upper row) and TG-treated cells (lower row) at 0 min (left) and after 60 min (right). **d** Statistical comparison of the MitoTracker fluorescence intensity change (ΔFI) over 60 min (corresponding to ROI) between control and TG-treated cells. *n* = 18–19. **e** Representative ER-Tracker staining in control (upper row) and TG-treated cells (lower row) at 0 min (left) and after 60 min (right). **f** Statistical comparison of the ER-Tracker fluorescence intensity change (ΔFI) over 60 min (corresponding to ROI) between control and TG-treated cells. *n* = 26–27. **g** Representative AM1-43 staining of endocytic vesicles from control (upper row) and TG-treated cells (lower row) at 0 min (left) and after 60 min (right). **h** Statistical comparison of the AM1-43 fluorescence intensity change (ΔFI) over 60 min (corresponding to ROI) between control and TG-treated cells. *n* = 14–16. Scale bar indicates 2 µm; ns not significant; **P < 0.01; The dashed lines show the respective ROIs (nucleus or cell).

The impact of TG on various intracellular compartments in RBL-1 cells was monitored with organelle-specific fluorescence dyes using confocal laser-scanning microscopy (CLSM) live cell imaging. Difference in fluorescence intensity (ΔFI) within 60 min was used to quantify TG-induced changes. Figure 1 shows representative images of HOECHST-fluorescence within the nucleus, MitoTracker labelling for evaluating mitochondrial integrity, ER-Tracker to evaluate ER distribution, and AM1-43 fluorescence for monitoring endocytosis along with statistical comparison of relative fluorescence between control and TG-treated RBL-1 cells. While HOECHST-fluorescence showed a significant decrease in TG-treated cells (**P < 0.01), MitoTracker-, ER-Tracker-, and AM1-43-fluorescence did not differ significantly between control and TG-treated cells over 60 min.

### Thapsigargin induces progressive ultrastructural disruptions in RBL-1 cells

**Fig. 2:**
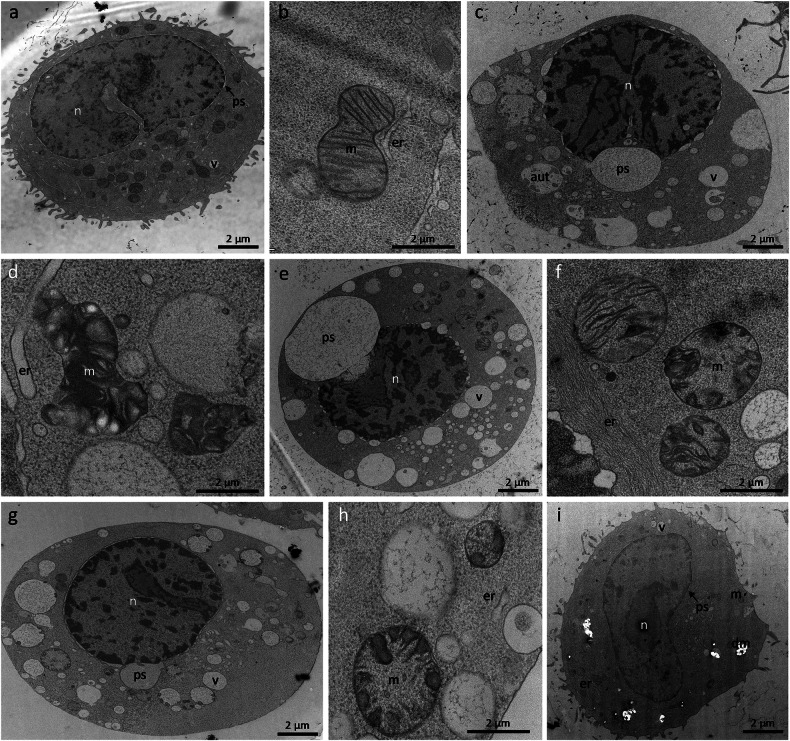
Arrangement and ultrastructure of organelles in RBL-1 cells before (control) and after treatment with TG and rescue of ultrastructural hallmarks via digoxin. **a** Overview image of an RBL-1 control cell with unremarkable distribution and morphology of intracellular organelles such as the nucleus (n), vesicles (v) and the perinuclear space (ps, arrow). **b** Detailed image of an RBL-1 control cell with the corresponding normal morphology of a mitochondrion (m) and ER (er). **c** Overview image of an RBL-1 cell treated with 2 µM TG for 10 min. The micrograph shows an enlargement of the perinuclear space, increased vacuolization (v) and autophagolysosomes (aut). Nuclear fragmentation and apoptotic bodies cannot be seen. Minor chromatin condensation is evident. **d** Detailed image of an RBL-1 cell treated with 2 µM TG for 10 min. Mitochondria appear enlarged and the cristae structure is degraded. The structure of the ER is recognizable, partially slightly enlarged although less abundant than in the controls. **e** Overview image of an RBL-1 cell treated with 2 µM TG for 30 min. The micrograph shows an enlargement of the perinuclear space and increased vacuolization. Nuclear fragmentation and apoptotic bodies cannot be seen. A slight chromatin condensation is evident. **f** Detailed image of an RBL-1 cell treated with 2 µM TG for 30 min. Mitochondria appear enlarged and the cristae structure is degraded. The structure of the ER is difficult to recognize and appears thinned out. **g** Overview image of an RBL-1 cell treated with 2 µM TG for 60 min. The micrograph shows an enlargement of the perinuclear space and increased vacuolization. Nuclear fragmentation and apoptotic bodies cannot be seen. A slight chromatin condensation is evident. **h** Detailed image of an RBL-1 cell treated with 2 µM TG for 60 min. Mitochondria appear enlarged and the cristae structure is degraded. The structure of the ER is difficult to recognize and appears thinned out in some areas. **i** RBL-1 cell after 60 min 2 µM TG treatment and subsequent rescue with 5 µM Digoxin (4 h) shows regular perinuclear space, regular vesicle abundance and ER morphology comparable to RBL-1 controls. Most mitochondria appear regularly shaped, similar to controls. Electron-poor, whitish areas show broken out, previously very electron-dense cristae structures of degrading mitochondria (dm).

While CLSM gives a first impression of the impact of TG on cell organelles, it does not replace transmission electron microscopy (TEM) for studying the ultrastructural effects of TG on RBL-1 cells. Initially, we analyzed the ultrastructure of untreated RBL-1 cells. An overview image of a control cell is presented in Fig. 2a, showcasing unremarkable distribution and morphology of intracellular organelles, including the nucleus (n), vesicles (v) and the perinuclear space (ps, arrow). A detailed image (Fig. 2b) further illustrates the normal morphology of mitochondria (m) and ER (er), confirming the baseline ultrastructural integrity of these organelles. Upon treatment with 2 µM TG for 10 min, significant ultrastructural changes were observed. The overview image (Fig. 2c) reveals enlargement of the perinuclear space, increased vacuolization (v) and the appearance of autophagolysosomes (aut). Notably, nuclear fragmentation and apoptotic bodies were not present, although slight chromatin condensation was evident. In addition to that, accumulation of small patches of condensed chromatin at the inner nuclear membrane was observed. Detailed examination (Fig. 2d) showed enlarged mitochondria with degraded cristae structure, while the ER remained recognizable, slightly enlarged but appeared less abundant compared to control cells. Further exposure to TG for 30 min resulted in more pronounced ultrastructural alterations. The overview image (Fig. 2e) continued to show an enlarged perinuclear space and increased vacuolization. Similar to the 10-min treatment, nuclear fragmentation and apoptotic bodies were absent, but slight chromatin condensation persisted. Detailed analysis (Fig. 2f) indicated enlarged mitochondria with severely degraded cristae structure. The ER structure was less discernible and appeared thinned out compared to control cells. Prolonged TG treatment for 60 min intensified the observed ultrastructural disruptions. The overview image (Fig. 2g) displayed further enlargement of the perinuclear space and extensive vacuolization, with consistent absence of nuclear fragmentation and apoptotic bodies, yet continued presence of slight chromatin condensation. The detailed image (Fig. 2h) revealed significantly enlarged mitochondria with extensively degraded cristae structure. The ER was challenging to recognize, appearing thinned out in several areas.

### Digoxin reverses TG-induced ER stress in RBL-1 cells

TEM micrographs revealed that 5 µM digoxin post-treatment (4 h) after 2 µM TG exposure (60 min) reversed most of the ultrastructural damage in RBL-1 cells (Fig. 2i). Rescue of TG-induced ER stress utilizing digoxin restored perinuclear space integrity, ER morphology, and vesicle abundance to control levels. Most mitochondria regained regular shape, although electron-lucent zones indicated residual cristae degradation in some mitochondria (Fig. 2i, dm).

### 3D TEM tomography confirms and highlights TG-induced ultrastructural alterations of organelles

**Fig. 3:**
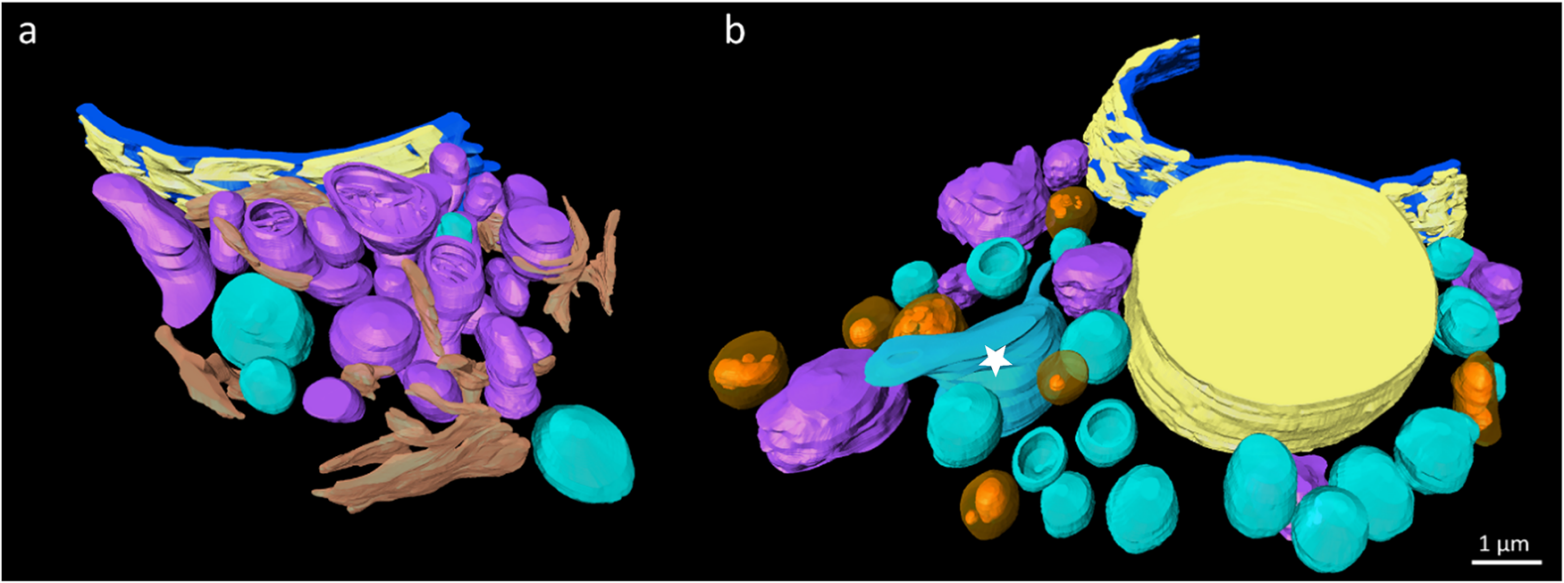
3D arrangement and structure of cell organelles of a control and TG-treated RBL-1 cell. **a** 3D TEM tomogram of a control RBL-1 cell with unaffected morphology and distribution of mitochondria (purple), ER (melon), vesicles (mint), nucleus (blue) and perinuclear space (yellow). **b** 3D TEM tomogram of an RBL-1 cell, 30 min after 2 μM TG treatment. Mitochondria appear enlarged, less abundant, with irregular surface structure. The ER is not visible in the thick tomography sections. Irregular vesicle shape (asterisk), vesicle number, vacuolization and number of autophagolysosomes (orange) appear to increase. The perinuclear space is greatly enlarged. Scale bar (1 µm) applies to both tomograms.

In addition to our 2D TEM investigations we provided a detailed spatial analysis of the intracellular architecture of RBL-1 cells before and after TG treatment, by implementing three-dimensional transmission electron microscopy (3D TEM) tomography on semi-ultrathin cell sections. The 3D TEM tomogram of a control RBL-1 cell (Fig. 3a; SVid. 1) exhibits a well-preserved and organized intracellular morphology. Mitochondria (purple), ER (melon), vesicles (mint), nucleus (blue) and the perinuclear space (yellow) are all visible and display normal distribution and morphology. This baseline 3D ultrastructure serves as a reference for comparison with TG-treated cells. Following treatment with 2 μM TG for 30 min, significant alterations in the 3D ultrastructure of RBL-1 cells were observed. The 3D TEM tomogram (Fig. 3b; SVid. 2) reveals pronounced changes, including enlarged but less abundant mitochondria with an irregularly shaped surface structure. Notably, the ER is not observable in the “thick” semi-ultrathin tomography sections. Additionally, there is an increase in the number of vesicles, indicative of enhanced vacuolization and an increase in the number of autophagolysosomes (orange). Few vesicles show an enlarged irregular shape (asterisk). The perinuclear space is greatly enlarged in specific regions, highlighting localized structural alterations.

### Statistical comparison of morphometric TEM analysis quantifies the ultrastructural qualitative results

**Fig. 4:**
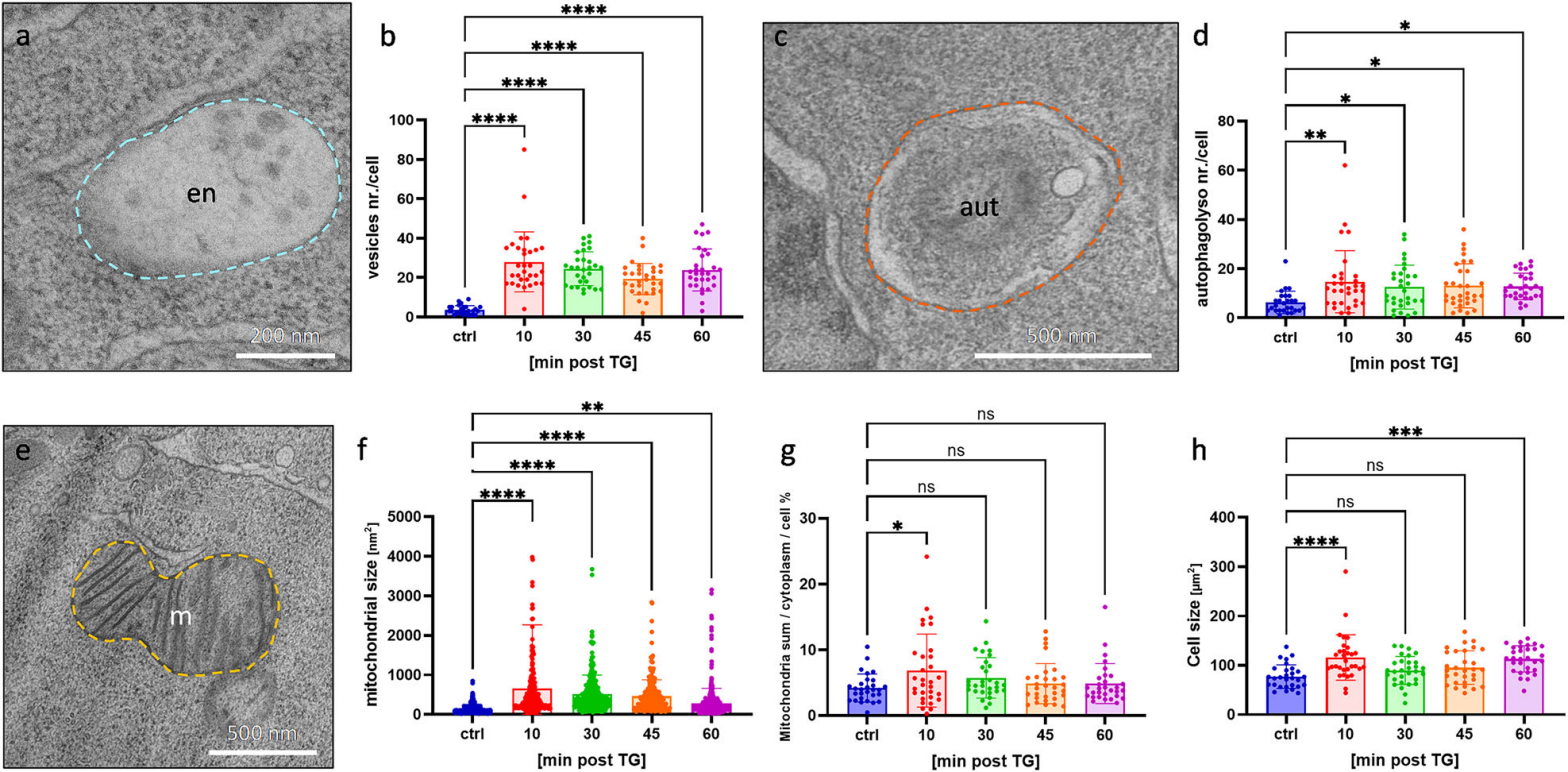
Statistical comparison of number of organelles per cell area and mitochondrial size in RBL-1 cells. **a** TEM micrograph with ROI (dashed in mint) of an RBL-1 control cell as a representative template for the statistical analysis of the vesicle number per cell in (**b**). Representation of vesicles per cell area corresponding to control cells and to cells treated with TG for 10, 30, 45 and 60 min of TG-treatment. *n* = 27–31. **c** TEM micrograph with ROI (dashed in orange) of an RBL-1 control cell as a representative template for the statistical analysis of the autophagolysosomal vesicle number per cell in (**d**). Comparison of RBL-1 control cells with cells after 10, 30, 45 and 60 min TG-treatment. *n* = 27-31. **e** TEM micrograph with ROI (dashed in yellow) of an RBL-1 control cell as a representative template for the statistical analysis of mitochondrial size in (**f**; *n* = 251–578) and total mitochondrial area per cytoplasm per cell in (**g**; *n* = 29-31) - RBL-1 control cells and cells after 10, 30, 45 and 60 min TG treatment times are compared. **h** TEM morphometric analysis of RBL-1 cell size in control cells and cells after 10, 30, 45 and 60 min TG treatment (*n* = 29–31). ns not significant; *P < 0.05; **P < 0.01; ***P < 0.001; ****P < 0.0001; The dashed lines show the respective ROIs. en endolysosomal vesicles, aut autophagolysosomal vesicles, m: mitochondria.

To quantify the morphological changes in RBL-1 cells induced by TG treatment, we performed a morphometric TEM analysis. This analysis focused on the number of general vesicles and autophagolysosomes, as well as mitochondrial size and area within the cytoplasm. An initial TEM micrograph of a control RBL-1 cell, highlighting the ROI in mint (Fig. 4a), served as a representative template for the statistical analysis of vesicle numbers. The quantification (Fig. 4b) showed a significant increase in the number of vesicles per cell following TG treatment for 10, 30, 45, and 60 min compared to control cells (****P < 0.0001). A TEM micrograph with the ROI highlighted in orange (Fig. 4c) was used to statistically analyze the number of autophagolysosomal vesicles per cell. The results (Fig. 4d) indicated a significant increase in autophagolysosomal vesicles in cells treated with TG for 10 (**P < 0.01), 30 (*P < 0.05), 45 (*P < 0.05), and 60 min (*P < 0.05) compared to controls. To assess mitochondrial changes, a TEM micrograph with the ROI highlighted in yellow (Fig. 4e) provided a basis for evaluating mitochondrial size and total mitochondrial area per cytoplasm. The analysis (Fig. 4f) revealed a significant increase in mitochondrial size in TG-treated cells at 10 (****P < 0.0001), 30 (****P < 0.0001), 45 (****P < 0.0001), and 60 min (**P < 0.01) compared to controls. Surprisingly, the total mitochondrial area per cytoplasm per cell was only significantly larger in TG-treated cells for 10 min compared to controls (*P < 0.05) but not significant for 30, 45 and 60 min TG treatment (Fig. 4g). TEM morphometric analysis also revealed a significant increase in RBL-1 cell size after 10 (****P < 0.0001) and 60 min (***P < 0.001) of TG treatment, with no significant changes observed at 30 and 45 min (Fig. 4h). Additional morphometric parameters are shown in SFig. 1a–f.

### Thapsigargin treatment shows minimal caspase activation and low Annexin V staining compared to staurosporine-mediated apoptosis

**Fig. 5:**
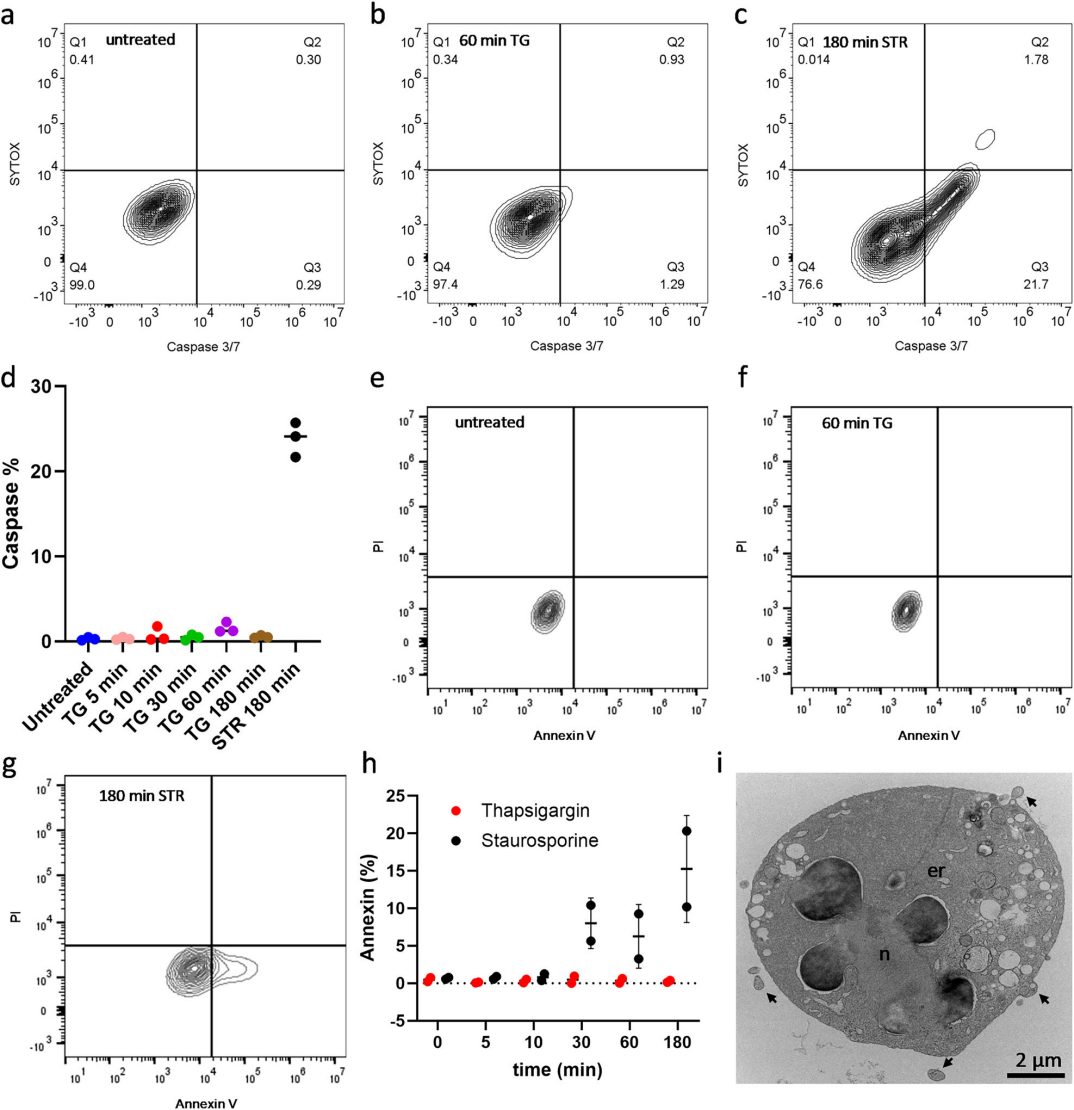
Cell death caspase - and Annexin 5 assays after TG and staurosporine treatment and representative TEM ultrastructure of RBL-1 cells, treated with staurosporine. **a** Representative contour plot image of the caspase 3/7 assay FACS data for untreated cells. **b** Contour plot image of the 60 min TG caspase 3/7 assay FACS data, representative for all TG treatments. **c** Representative caspase 3/7 contour plot image for staurosporine. **d** Caspase 3/7 cell death assay shows on the *X*-axis untreated, 5 min-, 10 min-, 30 min, 60 min - and 180 min TG-treated RBL-1 cells as well as the caspase positive staurosporine control (180 min). The *Y*-axis reflects the caspase 3/7 activity, which is increased upon staurosporine treatment (20-26%). All other treatments as well as untreated cells show a caspase 3/7 activity below 3%. **e** Representative contour plot images of the Annexin V assay for untreated, 60 min TG treatment (**f**) and 180 min staurosporine-treated cells (**g**). **h** Annexin V assay shows on the *X*-axis untreated (0 min), 5 min-, 10 min-, 30 min, 60 min and 180 min TG-treated and staurosporine-treated RBL-1 cells. The *Y*-axis reflects the Annexin V activity, which is increased upon 30 min, 60 min and 180 min staurosporine treatment (6–15%). All other treatments as well as untreated cells show an Annexin V activity below 2%. **i** TEM overview of an RBL-1 cell after staurosporine treatment (180 min), showing apoptotic features such as nuclear fragmentation and chromatin condensation (n) as well as swollen ER (er) and apoptotic bodies (arrows).

Since caspase activity is a biochemical hallmark for apoptosis [17, 29, 30], we evaluated caspase 3/7 activity in TG-treated cells and used staurosporine-treated RBL-1 cells as positive controls using a Caspase-3/7 Green Flow Cytometry Assay. As shown in Fig. 5(a-c), caspase activation was detected in staurosporine-treated cells and remained unchanged in control and TG-treated cells. While caspase activity in staurosporine-treated cells was between 20 and 26%, TG exposure for 5, 10, 30, 60 and 180 min showed caspase activity below 3% (Fig. 5d). Annexin V staining profiles from flow cytometry illustrated distinct responses across treatment conditions (Fig. 5e–h). Untreated cells (0 min, Fig. 5e) displayed baseline viability with minimal Annexin V signal. TG-treated cells (60 min, Fig. 5f) exhibited no notable shift in Annexin V staining compared to controls, a pattern consistent across all TG exposure durations. In contrast, staurosporine-treated samples (180 min, Fig. 5g) showed rightward shifts, reflecting elevated Annexin V binding. Quantitative analysis (Fig. 5h) confirmed staurosporine-induced Annexin V positivity (up to 15%) after 30–180 min, while untreated cells, TG-exposed RBL-1 cells (5–180 min), and short-duration staurosporine samples (5–10 min) all maintained staining levels below 2%. These results highlight the absence of significant phosphatidylserine externalization in TG-treated cells, contrasting with staurosporines’ time-dependent apoptotic effects. A representative TEM micrograph of an RBL-1 cell treated with staurosporine (180 min; Fig. 5i) displayed classical apoptotic features, including nuclear fragmentation, chromatin condensation, swollen ER and apoptotic bodies, confirming the apoptotic nature of cell death induced by staurosporine in RBL-1 cells.

### Morphological hallmarks of TG-induced cell death in RBL-1 cells do not match with apoptosis but with autosis

To elucidate the distinct morphological and physiological hallmarks associated with various forms of programmed cell death (PCD), we compared apoptotic PCD, non-apoptotic PCD (autosis as a representative) and the effects of TG-treatment on RBL-1 cells. Table 1 summarizes these hallmarks. Nuclear fragmentation, a characteristic feature of apoptotic PCD, was not observed in cells undergoing autosis or TG treatment. Similarly, apoptotic bodies were present in apoptotic PCD but absent in both autosis and TG-treated cells. ER swelling was noted in apoptotic PCD and described, albeit poorly, in autosis. TG treatment induced other significant ER changes/anomalies (such as thinning of the structure), differentiating it from apoptotic PCD. Chromatin condensation occurs prominently during apoptotic PCD. In contrast, chromatin condensation was described as mild for autosis which is also reflected by the results after TG treatment. Mitochondrial enlargements were observed across apoptotic PCD, non-apoptotic PCD and after TG treatment. Specifically, in autosis and TG treatment, mitochondrial degradation, particularly of the internal cristae, was evident and more pronounced than in apoptotic PCD. Vacuolization is not described as a hallmark in apoptotic PCD but for autosis and was also observed after TG treatment. Enlargement or ballooning of the perinuclear space was not described as a feature of apoptotic PCD. However, this was described as a notable hallmark for autosis and was also observed in TG-treated cells. An increase in autophagolysosomes, which is not recognized as an apoptotic hallmark, has been described as indicative of autosis. This increase was also analyzed in TG-treated RBL-1 cells. Conversely, cell shrinkage, a hallmark of apoptosis but not of autosis, was neither observed nor analyzed in TG-treated cells. Caspase dependence, a critical physiological marker of apoptotic PCD, was not described in autosis and was undetectable following TG treatment.

**Table 1:** Differentiation of known morphological and physiological hallmarks in apoptotic programmed cell death (apopt. PCD), autosis as a representative of non-apoptotic programmed cell death (non-apopt. PCD) and comparison with the results after TG treatment in RBL-1 cells.

Morphological hallmarks	Apopt. PCD	Non-Apopt. PCD (Autosis)	Thapsigargin
Nuclear fragmentation	Yes	No	No
Apoptotic bodies	Yes	No	No
ER swelling	Yes	Unclear	ER alterations/anomalies
Chromatin condensation	Yes	Mild chromatin condensation	Mild chromatin condensation
Mitochondrial alterations	Dilatation	Dilatation, degradation	Dilatation, cristae degradation
Vesicles and vacuolation	No	Yes	Yes
Ballooning of perinuclear space	No	Yes	Yes
Autophagolysosomal increase	No	Yes	Yes
Cell shrinkage	Yes	No	No

Physiological hallmark	Apopt. PCD	Non-Apopt. PCD (Autosis)	Thapsigargin
Caspase dependent	Yes	No	No

The following literature was used to create this table: [17, 25, 49, 50].

### Impact of transient and sustained cytoplasmic Ca^2+^ elevations on TG-induced cell death

**Fig. 6:**
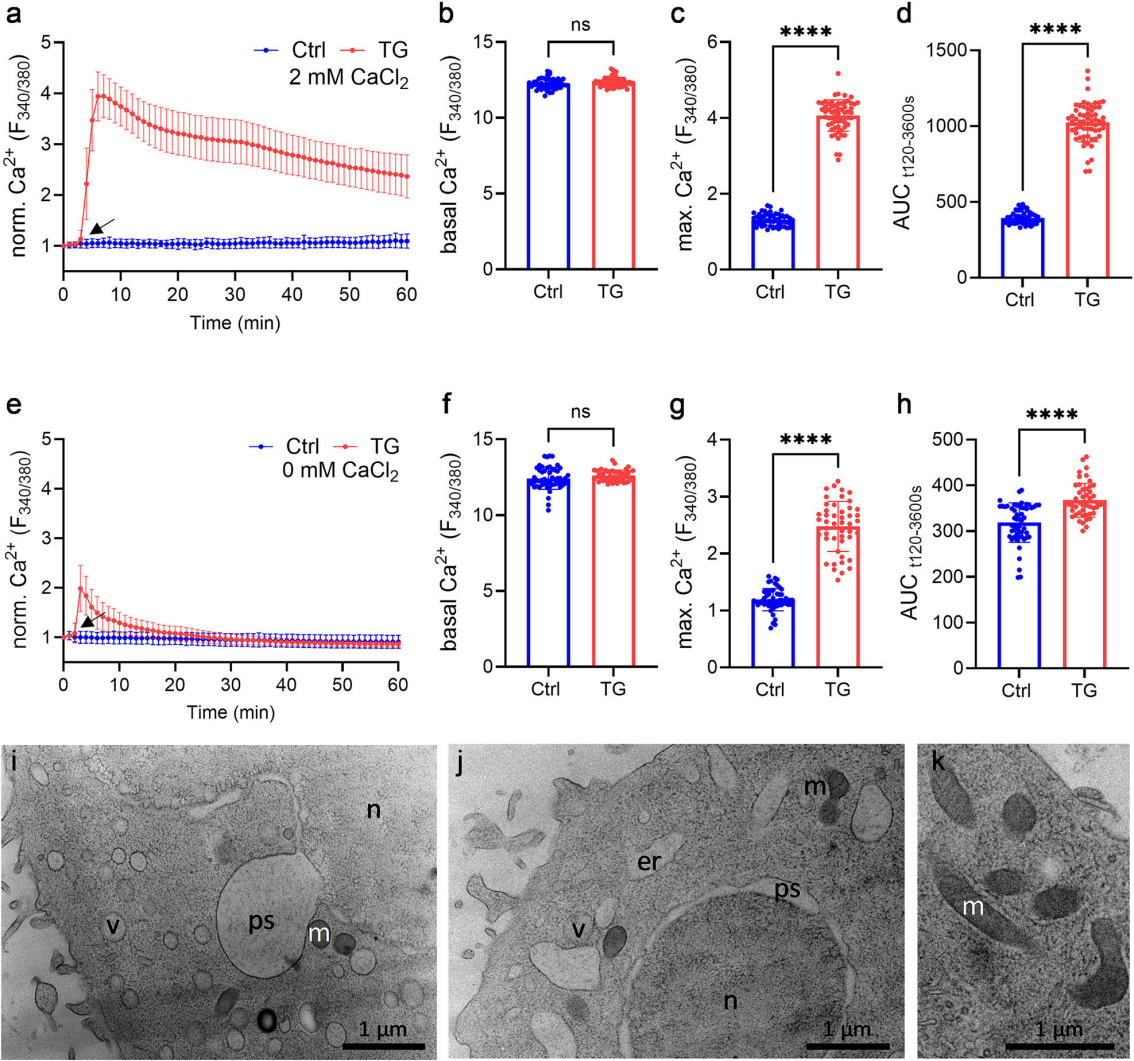
Ratiometric Ca^2+^ imaging of RBL-1 cells with and without extracellular Ca^2+^ with corresponding TEM micrographs. **a** Fura-2 based imaging of cytosolic Ca^2+^ concentration of RBL-1 cells in presence of 2 mM Ca^2+^. Cells were treated with 2 µM TG at the indicated time point (arrow) or left untreated, Ctrl (Ctrl, blue) and Thapsigargin (TG, red). Quantification of (**b**) basal Ca^2+^ (**c**) max Ca^2+^ influx and (**d**) area under the curve (AUC) of traces shown in (**a**), *n* = 51–61 cells. **e** Fura-2 based imaging of cytosolic Ca^2+^ concentration of RBL-1 cells in absence of Ca2+. Cells were treated with 2 µM TG at the indicated time point (arrow) or left untreated, Ctrl (Ctrl, black) and thapsigargin (TG, red). Quantification of (**f**) basal Ca^2+^ (**g**) max Ca^2+^ influx and (**h**) area under the curve (AUC) of traces shown in A, *n* = 48–54 cells. (**i-k**) Different cells, intracellular areas and magnifications of 2 µM TG-treated RBL-1 cells (60 min), without extracellular Ca^2+^ as a direct ultrastructural comparison to the Ca^2+^ imaging experiments. Cells show typical autotic hallmarks such as ballooning of the perinuclear space (ps) and vacuolization (v). In addition to that, cells show bloating of the ER (er) and regular mitochondrial (m) morphology.

To delineate the role of transient and sustained cytoplasmic Ca^2+^ on TG-induced autosis, we first compared the impact of the extracellular Ca^2+^ concentration on the TG-induced Ca^2+^ signal in RBL-1 cells. In the presence of 2 mM extracellular Ca²⁺, TG induced a rapid and sustained rise in cytosolic Ca²⁺ levels, peaking within 15–20 min and remaining elevated throughout the 60-min (Fig. 6a). Untreated control cells (Ctrl) exhibited stable baseline Ca²⁺ levels. Compared to control cells, in TG-treated cells the cytoplasmic Ca^2+^ level prior to TG application was similar to control cells (Fig. 6b). However, following application of TG increased the maximal cytoplasmic Ca^2+^ level 2.8-fold (Fig. 6c), and showed a 4.5-fold higher area under the curve (AUC) (Fig. 6d) (*p* < 0.001, *n* = 51–61 cells; 3 independent experiments). In nominally Ca²⁺-free medium, TG triggered a transient increase in cytoplasmic Ca^2+^ (Fig. 6e), showing a 2.5-fold increase in maximal cytoplasmic Ca²⁺ increase (Fig. 6g), and a 3.1-fold higher AUC cytoplasmic increase compared to control cells (Fig. 6h), indicating residual Ca²⁺ mobilization from intracellular stores (*p* < 0.001, *n* = 48–54 cells; 3 independent experiments). Interestingly, TG-stimulated ultrastructural changes, including ballooning of the nuclear envelope, an increase in the number of vesicles, degradation of mitochondria, and ER swelling, were also present following a transient TG-induced intracellular Ca^2+^ signal (Fig. 6i–k). However, in contrast to a sustained elevation in cytosolic Ca^2+^, due to extracellular Ca^2+^ influx, the ultrastructural changes were moderate. Again, apoptotic hallmarks such as nucleus fragmentation, chromatin condensation or apoptotic bodies were not observed.

### Thapsigargin induces autosis-like ultrastructural alterations in macrophages and mast cells

**Fig. 7:**
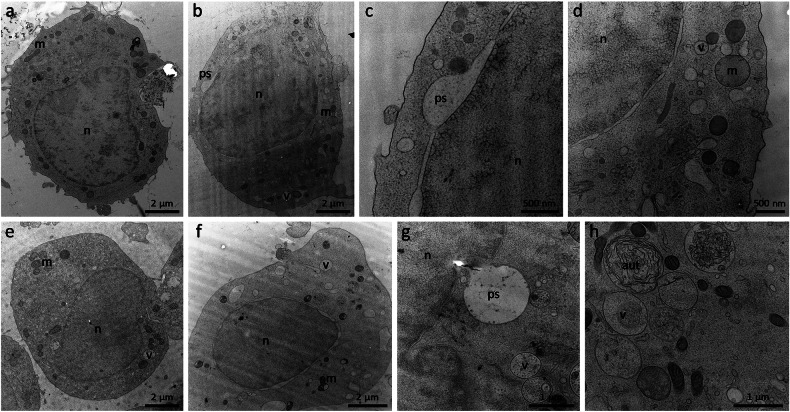
Ultrastructural alterations after 2 µM TG treatment (60 min) in murine macrophages and human mast cells. **a** J774 control cell with regular ultrastructure and in particular no noticeable alterations in nucleus (n) or mitochondria (m). **b** J774 cell treated with 2 µM TG (60 min) shows bloating of the perinuclear space (ps), increased vacuolation (v), and slightly enlarged mitochondria. **c** Higher magnification of the ballooning of the perinuclear space of a J774 cell after 60 min, 2 µM TG treatment. **d** Higher magnification of a J774 cell shows increased vacuolation and slightly enlarged mitochondria with a slight degradation of the mitochondrial cristae structure. **e** HMC1.2 control cell with regular ultrastructure without noticeable alterations in nucleus, mitochondria, or vesicles. **f** HMC1.2 cells treated with 2 µM TG (60 min) show swelling of the perinuclear space, increased vacuolation, and degrading mitochondria. **g** Higher magnification of ballooning of the perinuclear space and vesicles in an HMC1.2 cell after 60 min of 2 µM TG treatment. **h** Higher magnification of an HMC1.2 cell after 60 min, 2 µM TG treatment, shows increased vesicle abundance and increased autophagolysosomal (aut) vesicle structures.

TEM micrographs revealed distinct ultrastructural alterations in murine macrophages (J774) and human mast cells (HMC1.2) following 2 µM TG treatment for 60 min, consistent with autosis-like features mentioned above. Control J774 cells exhibited regular intracellular ultrastructure with intact nuclei and mitochondria (Fig. 7a). TG-treated J774 cells displayed perinuclear ballooning (Fig. 7b, c), pronounced cytoplasmic vacuolation, and mitochondrial enlargement with cristae degradation (Fig. 7b, d). Similarly, untreated HMC1.2 cells showed typical regular organelle architecture (Fig. 7e), while TG-treated HMC1.2 cells demonstrated marked perinuclear swelling/ballooning (Fig. 7f, g), increased abundance of autophagolysosomal vesicles, and partly degenerating mitochondria (Fig. 7f, h). Mitochondrial abnormalities in both cell types partly included cristae disorganization and matrix expansion. Notably, TG induced an increase in autophagolysosomal structures, especially in mast cells (Fig. 7h), characteristic of autosis.

## Discussion and conclusion

The present study elucidates a potential novel non-apoptotic, caspase-independent PCD mechanism induced by TG in RBL-1 cells, diverging from the classical apoptotic pathway. Through a series of advanced microscopy techniques, detailed morphometric analysis and cellular assays, we demonstrate that TG induces distinct alterations, suggesting an alternative, caspase-independent PCD modality similar to autosis, rather than traditional apoptosis.

Apoptosis is a highly regulated cell death process that occurs in multicellular organisms, playing a crucial role from embryogenesis to aging [15, 31]. Its deregulation can lead to various pathological conditions, including cancer and neurodegenerative diseases [32, 33]. One of the hallmarks of human cancers is the intrinsic or acquired resistance to apoptosis. This resistance may contribute to therapy resistance, as many anticancer therapies activate apoptotic processes in cancer cells [34–36]. Understanding the mechanisms of apoptosis evasion by cancer cells is of central importance in drug development, as is a better understanding of alternate PCD pathways.

Here we find distinct morphological and physiological features following TG treatment. The ballooning of the perinuclear space emerged as a predominant and exclusive morphological hallmark, consistently attributed to autosis [25, 26, 37]. This feature was frequently noted across all incubation periods following TG treatments in our experiments. This morphological feature could also be attributed to the so-called “nuclear envelope budding” (NEB), which seems to be responsible for transporting large ribonucleotides from the cell nucleus into the cytoplasm [38, 39]. However, the enlargement of the cell nucleus described here appears relatively “electron-thin” in the TEM micrographs, which is more likely a vacuole-like structure with probably very diluted nuclear material. In connection with the balloon-like morphology and size, the term “ballooning” defines this ultrastructural phenotype best. However, no studies have been conducted to date to investigate a connection between NEBs and ballooning of the perinuclear space.

Contrary to the expectations of apoptosis, nuclear fragmentation was absent in TG-treated cells, as evidenced by TEM images and CLSM experiments with nuclear staining. In contrast, staurosporine-treated cells exhibited clear nuclear fragmentation, a well-studied indicator of apoptosis [17, 40]. Apoptotic bodies, another hallmark of apoptosis [17], were identified in staurosporine-treated cells but were notably absent in TG-treated cells, both in TEM images and ESID images from CLSM experiments. ER swelling, typically associated with apoptotic cells [17], was observed in staurosporine-treated cells. Interestingly, TG-treated cells exhibited slight ER swelling within the initial 10 min of treatment, with ER structures appearing to diminish after 30 min in RBL-1 cells. At higher TEM magnification, ER-like structures with a narrow, contracted lumen were occasionally observed, suggesting partial persistence of the ER. CLSM experiments with ER labelling indicated no overall change in intensity, implying a potential compensatory extension of ER structures, which may explain their apparent “disappearance” at lower magnifications, as it was previously described for autosis [25].

Chromatin condensation, a common apoptotic feature [17], was confirmed in staurosporine-treated cells. TG-treated cells, however, showed only mild chromatin condensation, aligning with characteristics of autosis [25–27]. Our TEM experiments revealed that TG-treated cells had enlarged mitochondria with degraded inner membranes. Morphometric TEM analyses of RBL-1 cells showed a slight increase in the total mitochondrial area relative to the cytoplasm after 10 min of TG treatment, but no significant difference after 30 min compared to controls, consistent with CLSM results and in align with known autotic features [25, 26]. An increase in intracellular vesicles, characteristic of autosis but not apoptosis [17, 25, 26], was frequently observed following TG treatment. Given the lack of significant changes in endocytosis in CLSM experiments, these vesicles likely originated intracellularly, possibly from the ER or Golgi apparatus. The presence of increased autophagolysosomes, a feature of both autophagy and autosis [25], was confirmed by our 2D, 3D and morphometric TEM analyses. Lastly, cell shrinkage, a phenomenon observed in apoptotic cells [17], was not evident in TG-treated cells. Instead, morphometric data indicated either no change or even a significant increase in cell area.

To underline the autosis-like ultrastructural characteristics after TG treatment in RBL-1 cells, an autosis rescue experiment was performed, utilizing digoxin [25, 28]. Indeed, digoxin attenuated TG-induced ER stress, consistent with its inhibition of Na^+^/K^+^-ATPase (NKA) [28]. This might stabilize Ca^2+^ flux and counteract the SERCA inhibitory effects. The partial mitochondrial recovery suggests that digoxin preferentially restores ER homeostasis rather than repairing mitochondrial dysfunction. These results highlight the potential of digoxin to mitigate ER stress-mediated autosis. However, partly persistent cristae damage underscores the need for combinatorial strategies to target mitochondrial ER crosstalk.

Overall, the morphological features observed in TG-treated RBL-1 cells align closely with characteristics of autosis rather than apoptosis, providing valuable insights into the distinct cellular responses to TG treatment. This is further supported by our caspase-dependent cell death and Annexin V assays. The caspase assay revealed significantly lower caspase activity in TG-treated cells compared to the markedly high caspase activity observed in staurosporine-treated cells, as it was reported before [41, 42]. This is also supported by a significantly lower Annexin V staining in TG treated RBL-1 cells compared to staurosporine treated cells. This thereby underscores the distinct pathways of cell death induced by TG and staurosporine, aligning with our morphological observations and reinforcing the characterization of TG-induced cell death as non-apoptotic.

Our Ca^2+^ imaging experiments demonstrated that TG-induced cytosolic Ca²⁺ elevation originates primarily from indirect ER Ca²⁺ release, with extracellular Ca²⁺ entry amplifying the sustained phase. The persistence of autotic features in Ca²⁺-free medium supports ER-derived Ca²⁺ as the critical driver of autosis initiation, while sustained influx of extracellular Ca²⁺ intensifies the magnitude of downstream effects. In accordance with that, ultrastructural electron microscopic analysis of cells treated with TG in Ca²⁺-free medium demonstrated similar hallmark features of autosis, including ballooning of the perinuclear space and vacuolization. However, slight differences in the effects on mitochondria and ER are observed in TG treatment without extracellular Ca^2+^, such as increased bloating of ER structures. Mitochondria, on the other hand, show fewer ultrastructural changes compared to TG treatment with extracellular Ca^2+^. Both can be linked to the lower intracellular cytosolic Ca^2+^ concentration due to the lack of extracellular Ca^2+^ influx [43, 44]. This furthermore confirms that ER-derived Ca²⁺ mobilization is critical for autosis induction, while extracellular Ca²⁺ entry via SOCE amplifies some of the described autotic ultrastructural effects.

Rat basophilic leukaemia cells were selected as a main cell model system for their dual relevance as a basophilic cancer line derived from innate immune cells, providing a unique platform for cancer- and immune cell research as well as their precise ultrastructural characterization [45–47]. To ensure translational breadth, we extended analyses to murine J774 macrophages and human HMC1.2 mast cells, encompassing evolutionarily conserved innate immune effectors and human translational models. Consistent autotic features (e.g., vacuolization, ER stress-linked perinuclear alterations) across these cell types - despite minor mitochondrial morphological variations—support TG-induced autosis as a conserved mechanism independent of cellular origin. Structural changes align with TG’s known effects: ER calcium depletion via SERCA inhibition, disrupted Ca^2+^ homeostasis, and autophagic flux impairment. Partial mitochondrial swelling and cristae breakdown further corroborate energy crisis pathways. While cell-type-specific subcellular variations suggest differential susceptibility, our findings position TG-driven ER stress and Ca^2+^ dysregulation as central mediators of autosis in innate immune cells. Ultrastructural evidence points to a broad TG effect across immune and cancer cells, though further validation is required to confirm universal applicability.

Regarding the methodological spectrum of this study, CLSM live cell imaging serves as a powerful tool for investigating organellar behaviour due to its advanced imaging capabilities. However, its resolution limitations necessitate the use of ultrastructural 2D and 3D TEM to achieve comprehensive correlation and validation of findings, thereby integrating the detailed ultrastructural insights of TEM with the dynamic imaging afforded by CLSM.

The unique morphological and physiological characteristics of TG-induced PCD may be explored for therapeutic strategies. The ability of TG to induce a non-apoptotic, caspase-independent form of PCD may be particularly advantageous in targeting cancer cells that have developed resistance to apoptotic mechanisms. This aligns with recent findings on the anticancer activity of TG and its prodrugs, which exploit the vulnerability of cancer cells to ER stress and UPR [1, 8, 10]. Moreover, the distinct features of TG-induced PCD, including mitochondrial degradation and autophagic vesicle accumulation, suggest that combination therapies targeting both apoptotic and non-apoptotic pathways could enhance therapeutic efficacy and overcome resistance mechanisms.

A potential limitation of our study is the confinement of TG incubation to a 3-hour period. Exploring whether apoptotic induction occurs beyond this time frame remains an open question. Future investigations extending the incubation period could provide valuable insights into the possible transition from non-apoptotic to apoptotic forms of programmed cell death, a process that has yet to be elucidated.

Our study provides compelling evidence that TG induces a potentially novel non-apoptotic, caspase-independent form of PCD in RBL-1 cells, characterized by distinct morphological and ultrastructural changes. This alternative PCD modality shares features with autosis, offering new insights into the cellular response to ER stress and the potential for therapeutic exploitation in cancer and other diseases. As our understanding of non-apoptotic PCD pathways expands, TG and its respective precursor compounds remain valuable tools for both basic research on intracellular Ca^2+^ homeostasis and clinical applications, promising new avenues for effective and targeted therapies.

## Material and methods

### Cell line and in vitro experiments

Adherent rat basophilic leukaemia cells (RBL-1; provided by AG Zierler, Walther Straub Institute of Pharmacology and Toxicology, LMU Munich) were used as cell line for all experiments. The cells were grown in Dulbecco’s Modified Eagle’s Medium (DMEM; Gibco, Thermo Fisher Scientific, Waltham, USA) with 10% fetal bovine serum (FBS; Gibco; Thermo Fisher Scientific, Waltham, USA) in T-75 cell culture flasks (Sigma Aldrich, St. Louis, USA). Ambient parameters were at 5% CO_2_ and 37 °C. In addition, the macrophage cell line J774 (provided by AG Klugbauer, Institute of Experimental and Clinical Pharmacology and Toxicology, University of Freiburg; DMEM with 10% FBS) and the human mast cell line HMC1.2 (Sigma-Aldrich, St. Louis, USA; IMDM with 10% FBS) were used for ultrastructural key experiments. For all experiments, cells were maintained in culture for at least one week and a passage level of 10 was never exceeded. The sesquiterpene-lactone thapsigargin (TG; Fisher Scientific GmbH, Schwerte, Germany) was used to inhibit SERCA (Sarco/Endoplasmic Reticulum Ca^2+^ ATPase) and to subsequently trigger programmed cell death. A concentration of 2 µM and an incubation time of 10 min was initially used, based on earlier physiological experiments [45] and was extended to 5, 30, 45 and 60 min according to respective experiments.

### Confocal laser scanning microscopy (CLSM) live cell imaging

RBL-1 cells were seeded with a cell density of approx. 150,000–200,000 cells in 35 mm ibidi® µ-dishes (ibidi GmbH, Gräfelfing, Germany) and were incubated overnight in 800 µl DMEM at 5% CO_2_ and 37 °C. Controls and cells treated with 2 µM TG were used for microscopic analyzation. The following dyes were used for live-cell imaging: 8 μM AM1-43 (BIOZOL, Eching, Germany) was used as a marker for endocytosis (5 min dark incubation in PBS at 5% CO_2_ and 37 °C; *n* = 14–16). 1 µM ER-Tracker™ Blue-White DPX (Molecular Probes, Eugene, USA) was used as staining for the ER (30 min dark incubation in serum-free DMEM at 5% CO2 and 37 °C; *n* = 26–27). 100 nM MitoTracker® Orange CMTMRos (Thermo Fisher Scientific, Waltham, USA) was used as staining for mitochondria (30 min dark incubation in serum-free DMEM at 5% CO_2_ and 37 °C; *n* = 18–19). 10 µM HOECHST 33342 (Thermo Fisher Scientific, Waltham, USA) was used as staining for the nucleus (10 min dark incubation in PBS at 5% CO_2_ and 37 °C; *n* = 25). After each staining, the cells were washed twice with phosphate-buffered saline (PBS; Sigma-Aldrich, St. Louis, USA) and microscopic analysis was also performed in PBS. A Zeiss LSM 900 Airyscan 2 (Carl Zeiss Meditec AG, Oberkochen, Germany) was used for laser scanning microscopy. The samples were analyzed with a 63×/1.4-oil objective (Carl Zeiss Meditec AG, Oberkochen, Germany) and the Airyscan 2 detector (Carl Zeiss Meditec AG, Oberkochen, Germany). Controls were compared with 2 µM TG-treated cells and fluorescent intensity profiles were created over a 60 min period to exclude intensity peaks occurring before 60 min. For the TG-treated cells, an image was taken before TG addition (0 min). To calculate the fluorescence change ΔFI, the mean fluorescence of a cell after 60 min was determined for both controls and TG-treated cells and the initial value (0 min) was subtracted from the same cell. Excitation and emission wavelengths for HOECHST were 405/455, for AM1-43 488/579, for MitoTracker 561/575 and for ER-Tracker 405/557. Airyscan images were processed for image analysis and were analyzed with ZEN 3.4 blue software (Carl Zeiss Meditec AG, Oberkochen, Germany) and the intensity change ∆FI in specific ROIs (region of interest) was used for graphs and statistical analysis. All experiments were repeated 3 times independently.

### Transmission electron microscopy

For the preparation of 2D TEM, 3D TEM tomography and morphometric TEM analysis, RBL-1 cells (controls and cells treated with 2 µM TG for 10, 30, 45 and 60 min) as well as 2 µM TG (60 min) without extracellular Ca^2+^, 20 µM staurosporine (3 h), 5 µM Digoxin (4 h, after 1 h of 2 µM TG) treated cells (only for 2D TEM) and J774 and HMC1.2 cells (untreated and TG treated for qualitative 2D TEM) were first transferred to specific sample holders (Leica Microsystems, Vienna, Austria) for high pressure freeze fixation (HPF). The subsequent HPF implementation was performed in a Leica EM ICE device (Leica Microsystems, Vienna, Austria). Care was taken to ensure that a cooling rate of at least 17,500 °C per second and a pressure rate of at least 2040 bar were achieved. The freeze-fixed cells were then transferred to a pre-chilled automatic freeze substitution unit (Leica EM AFS2; -90 °C; Leica Microsystems, Vienna, Austria) and kept at constant temperature for 25.5 h. After that, temperature increased as follows: from −90 °C to −85 °C in 8 h, from −85 °C to −60 °C in 30 min, kept at −60 °C for 30 min, from −60 °C to −30 °C in 1 h, kept at −30 °C for 1 h, from −30 °C to 0 °C in 1 h and from 0 °C to 15 °C in 1 h. Afterwards cells were further warmed up and stabilized at room temperature (approx. 20 °C). The cryosubstitution medium used for these steps contained 1% osmium tetroxide (OsO_4_) in anhydrous acetone. After cryosubstitution, the samples were washed three times with anhydrous acetone and three times with propylene oxide and then embedded in epoxy resin (medium quality; Agar Scientific, Essex, UK). The embedding took place in Beem capsules (Agar Scientific, Essex, UK), since this ensured that the cells could be collected in the lower tip of the capsules. This in turn was crucial for the correct execution of the further sample preparation, in particular for the sectioning process on the ultramicrotome (see below). Before that, the cells had to be polymerized at +70 °C for 24 h. After this step, ultra-thin sections for conventional 2D TEM analysis (~60 nm) and sections for 3D TEM tomography (~800–1000 nm) were prepared on a Leica UC7 ultramicrotome (Leica Microsystems, Vienna, Austria). Sections were collected on Formvar-coated copper mesh grids (Science Services GmbH, Munich, Germany) for the 2D applications and on parallel grids for 3D applications. The 2D TEM micrographs of RBL-1-, J774- and HMC1.2 cells were recorded at a Talos L120C G2 STEM (Thermo Fisher Scientific, Waltham, USA) at an acceleration voltage of 120 kV. TEM images were recorded using Velox software (version 3.10.0.1130-5d766716c0; Thermo Fisher Scientific, Waltham, USA) and were analyzed using Image J [48]. The 3D TEM tomograms were also recorded at 120 kV using tilt images between the maximum angles of -60 ° and +60 ° with an increment of 3°. Tomo software (version 5.17.0.6930; Thermo Fisher Scientific, Waltham, USA) was used for the acquisition of the tilting images. Pre-alignment and conversion were carried out using IMOD (Mastronarde, Boulder, University of Colorado, USA) and for the post-alignment, segmentation and 3D reconstruction AMIRA™ (Thermo Fisher Scientific, Waltham, USA) was used.

### Morphometric TEM analysis

RBL-1 control cells and cells treated for 10, 30, 45 and 60 min with 2 µM of TG were used for TEM morphometric analysis. Ultrastructural data were measured using ImageJ [48] and specific areas (ROIs) were used for analysis: number of vesicles per cell (*n* = 27–31), number of autophagolysosomes per cell (*n* = 27–31), mitochondrial area (nm^2^; *n* = 251–578) and mitochondrial area in relation to total cytoplasm area (%; *n* = 29–31). For the latter morphometric unit, the area of the cell minus the nucleus was calculated to calculate the total cytoplasm area. This area was then expressed as a percentage of the total mitochondrial area in each individual cell:

$${Mito\; sum}/{Cytoplasm}/{Cell}[ \% ]=\frac{\Sigma\, A\,{mito}\,{{{\mu }}m}^{2}}{\left(A\,{cell}\,{{{\mu }}m}^{2}-A\,{nucelus}\,{{{\mu }}m}^{2}\right)}{{\cdot }}100$$

All morphometric measurements were performed by at least two independent persons to exclude subjective/biased measurements.

### Caspase 3/7 cell death assay

Cells were treated with 2 μM TG for 5, 10, 30, 60 and 180 min. Staurosporine (20 μM for 180 min) was used as a control for caspase activation. After treatment, the cells were incubated with the CellEvent™ Caspase-3/7 Green Flow Cytometry Assay Kit (Thermo Fisher Scientific, Waltham, USA, Cat. No. C10427) in complete growth medium for 30 min. For the caspase 3/7 cell death assay, cells were seeded in 6-well plates (Sarstedt AG & Co. KG, Nümbrecht, Germany) at a density of 500,000 cells per well and allowed to adhere for 24 h. For the assay, adhered cells were collected and the CellEvent™ Caspase-3/7 Green Flow Cytometry Assay Kit was used to measure caspase 3/7 activity (3 independent experiments). The reagent consists of a four amino acid peptide (DEVD) conjugated to a nucleic acid binding dye. This cell-permeant substrate is intrinsically non-fluorescent, because the DEVD peptide inhibits the ability of the dye to bind to DNA. After activation of caspase-3 or caspase-7 in apoptotic cells, the DEVD peptide is cleaved, enabling the dye to bind to DNA and produce a bright, fluorogenic response with absorption/emission maxima of ~511/533 nm. In combination with SYTOX™ AADvanced™ Dead Cell Stain, apoptotic cells can be clearly differentiated from live and necrotic cells. The cells were then imaged and analyzed using a CytoFLEX V5-R3-B5 (Beckman Coulter GmbH, Krefeld, Germany) to detect the green fluorescence signal indicative of caspase 3/7 activity. The results were analyzed and gating strategies (SFig. 2) were defined using FlowJo™ software (Becton, Dickinson and Company, Ashland, USA) and were depicted using GraphPad Prism 10 (v.10.2.3).

### Annexin V assay

RBL-1 cells were seeded at a density of 500,000 cells into 6-well plates and grown overnight at 37 °C. Cells were treated with 2 μM TG or 20 μM staurosporine for 5, 10, 30, 60 and 180 min, or left untreated. Adhered cells were collected and stained on ice for Annexin-V-APC (Biolegend, 640919) and Propidium Iodide (Miltenyi, 130-093-233) in Annexin V staining buffer including (in mM): 10 HEPES pH 7.4, 140 NaCl, 2.5 CaCl_2_. The cells were then imaged and analyzed using a CytoFLEX V5-R3-B5 (Beckman Coulter GmbH, Krefeld, Germany; 2 independent experiments). The results were analyzed and gating strategies (SFig. 2) were defined using FlowJo™ software (Becton, Dickinson and Company, Ashland, USA) and were depicted using GraphPad Prism 10 (v.10.2.3).

### Ca^2+^ imaging

RBL-1 cells were seeded with a cell density of approx. 100,000 cells in 35 mm ibidi® µ-dishes (ibidi GmbH, Gräfelfing, Germany) and were incubated overnight in 800 µl DMEM at 5% CO_2_ and 37 °C. Cells were loaded with 3 μM Fura-2 AM and 0.05% Pluronic®F-127 (Invitrogen, Thermo Fisher Scientific, Waltham, USA) in imaging buffer for 15 min at 37 °C. Ca^2+^ and Mg^2+^-free HBSS imaging buffer was supplemented with (in mM): 2 CaCl_2_, 0.4 MgCl_2_, 11 glucose and 5 HEPES, pH 7.2. After staining, cells were washed once with imaging buffer to remove excess dye, following an incubation for 5 min at 37 °C in imaging buffer. Cells were subsequently used for imaging. Time lapse images were acquired every 10 s on an AnglerFish imaging system (Next Generation Fluorescence Imaging/ NGFI, Graz, Austria). Upon recording of baseline signals for 2 min, cells were treated with 2 µM TG or left untreated. Viable cells were identified by their ionomycin response at the end of the measurement and were analyzed with Fiji (*n* = 51-61). For ER depletion experiments, all buffers were prepared without CaCl_2_, experimental procedure was identical as stated above (*n* = 48-54).

### Statistical analysis

Data are presented as means ± SD unless otherwise indicated in the figure legends. The statistical analysis was elaborated with GraphPad Prism 10 (v.10.2.3). Significance was determined using an ordinary ANOVA (including Brown-Forsythe and Bartlett’s test) with Dunnett’s multiple comparisons test for morphometric TEM analysis and unpaired t-test for CLSM live cell imaging and Ca^2+^ imaging data. The degree of significance in *P*-values is marked with asterisks (ns: not significant; *P < 0.05; **P < 0.01; ***P < 0.001; ****P < 0.0001).

## Supplementary information

Supplementary Figure 1

Supplementary Figure 2

Supplementary Data

Supplementary Video 1

Supplementary Video 2

## Data Availability

Data and materials may be requested by the corresponding author.
