# Supplementary figures and images for: Thapsigargin triggers a non-apoptotic, caspase-independent programmed cell death in basophilic leukaemia cells
Source: Cell Death Discov. 2025 Jul 8;11:313. doi: 10.1038/s41420-025-02602-w (PMC12238333; doi:10.1038/s41420-025-02602-w)

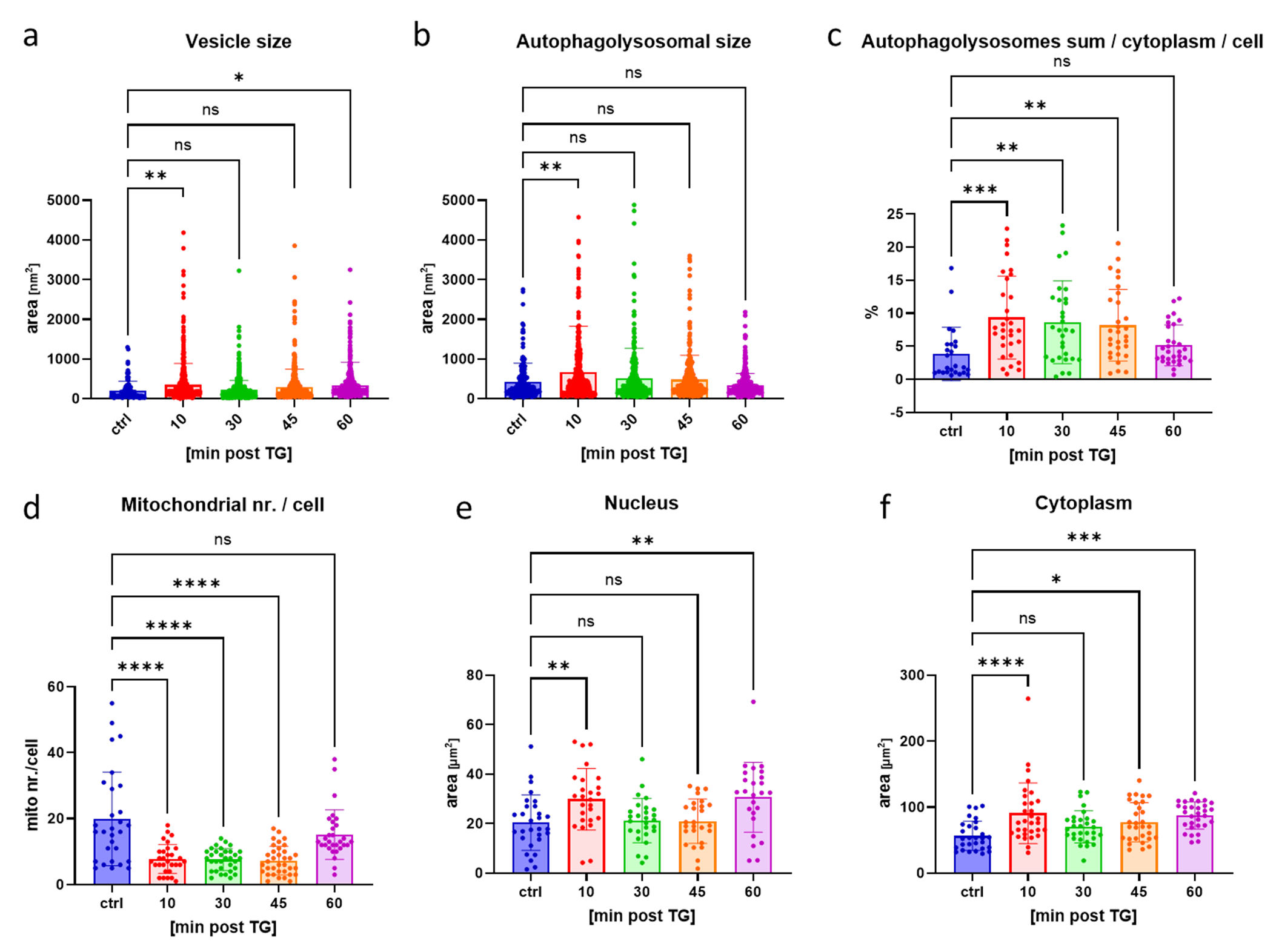

Supplement: Supplementary file 1 — Supplementary Figure 1 [file 41420_2025_2602_MOESM1_ESM.jpg]

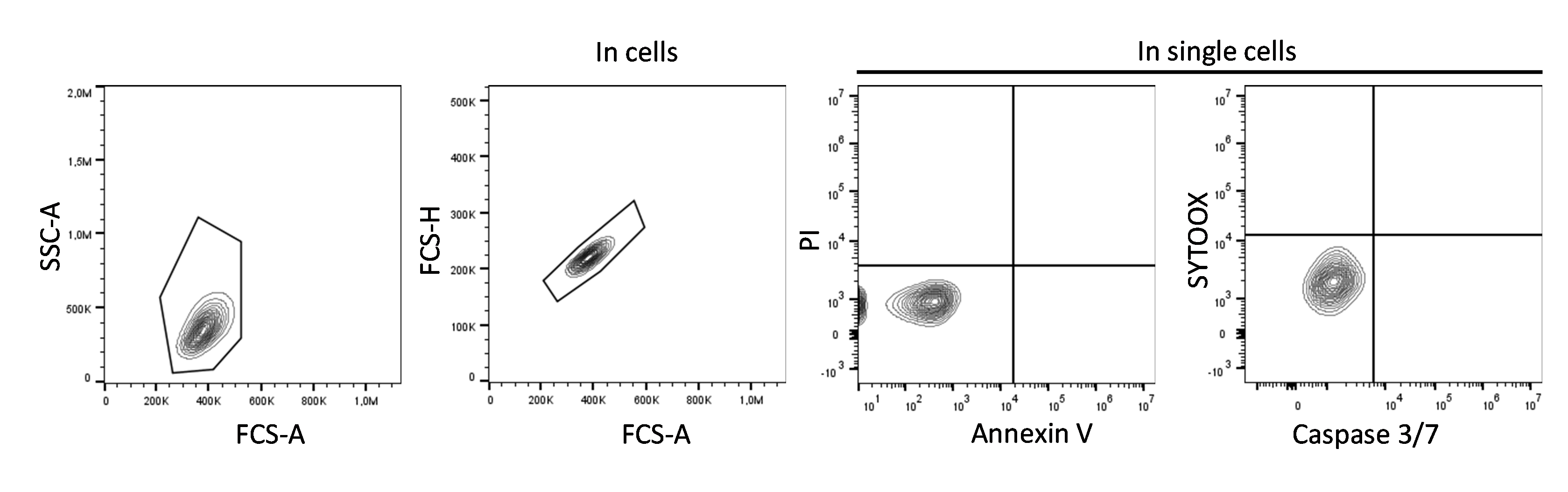

Supplement: Supplementary file 2 — Supplementary Figure 2 [file 41420_2025_2602_MOESM2_ESM.png]
