# Supplementary material for: Thapsigargin triggers a non-apoptotic, caspase-independent programmed cell death in basophilic leukaemia cells
Source: Cell Death Discov. 2025 Jul 8;11:313. doi: 10.1038/s41420-025-02602-w (PMC12238333; doi:10.1038/s41420-025-02602-w)
Supplement: Supplementary file 3 — Supplementary Data [file 41420_2025_2602_MOESM3_ESM.docx]

**Supplementary data**

**SFig. 1 Morphometric TEM analysis of different organelles in RBL-1 control cells and cells treated with TG. (a)** Morphometric TEM analysis of vesicle size/area [nm^2^] in RBL-1 control cells and cells after 10, 30, 45 and 60 min TG treatment times are compared. n=95-866. **(b)** Morphometric TEM analysis of autophagolysosomal size/area [nm^2^] in RBL-1 control cells and cells after 10, 30, 45 and 60 min TG treatment times are compared. n=170-456. **(c)** Morphometric TEM analysis of autophagolysosomes per cytoplasm per cell [%] in RBL-1 control cells and cells after 10, 30, 45 and 60 min TG treatment times are compared. n=25-31. **(d)** Morphometric TEM analysis of mitochondrial number per cell in RBL-1 control cells and cells after 10, 30, 45 and 60 min TG treatment times are compared. n=29-35. **(e)** Morphometric TEM analysis of nucleus size/area [µm^2^] in RBL-1 control cells and cells after 10, 30, 45 and 60 min TG treatment times are compared. n=25-29. **(f)** Morphometric TEM analysis of cytoplasm size/area [µm^2^] in RBL-1 control cells and cells after 10, 30, 45 and 60 min TG treatment times are compared. n=29-31. ns: not significant; *: P < 0.05; **: P < 0.01; ***: P < 0.001; ****: P < 0.0001

**SFig. 2 Gating strategy for Annexin V and Caspase 3/7 assay**

Representative FACS plots and gating strategy for Annexin V and Caspase 3/7 staining of RBL-1 cells shown for control.

**SVid. 1 360° video of a 3D TEM tomography reconstruction of an intracellular section of an RBL-1 control cell for comprehensive representation.** Unaffected morphology and distribution of mitochondria (purple), ER (melon), vesicles (mint), nucleus (blue) and perinuclear space (yellow).

**SVid. 1 360° video of a 3D TEM tomography reconstruction of an intracellular section of an RBL-1 cell 30 min after 2 µM TG treatment for comprehensive representation.** Mitochondria (purple) appear enlarged, with irregular surface structure. The ER is not visible in the “thick” tomography sections. Vesicle (mint) number, vacuolization and number of autophagolysosomes (orange) appear to increase. The perinuclear space (yellow) is greatly enlarged.
